# Efficacy of auriculotherapies for primary insomnia: A systematic review and network meta-analysis

**DOI:** 10.1097/MD.0000000000048357

**Published:** 2026-04-24

**Authors:** Zhaolong Luo, Jiao Shen, Xiangqin Chen, Qiong Zhang, Jiangyan Xiong

**Affiliations:** a Nursing College of Chongqing Three Gorges Medical College, Wanzhou, Chongqing, China; b Nursing College of Guizhou University of Traditional Chinese Medicine, Guiyang, Guizhou, China; c Department of Pediatrics, Chongqing University Three Gorges Hospital, Wanzhou, Chongqing, China.

**Keywords:** auriculotherapy, insomnia, network meta-analysis, randomized controlled trials, traditional Chinese medicine

## Abstract

**Background:**

Auriculotherapy has been used extensively for insomnia in traditional Chinese medicine due to its high effectiveness and few adverse effects. However, there are several auriculotherapy approaches, and it’s still uncertain which is best for treating primary insomnia (PI). The purpose of this study is to determine the best auriculotherapy strategy for treating PI.

**Methods:**

A comprehensive search was conducted across 8 databases to identify randomized controlled trials investigating the use of auriculotherapy for the treatment of PI. The search period spanned from database inception through July 28, 2025. The observation group received either single or combined auriculotherapy, while the control group was given another type of auriculotherapy, oral eszopiclone, sham stimulation, or usual care. The primary outcome was the Pittsburgh Sleep Quality Index, and the secondary outcome was safety assessments. Methodological quality was assessed with the Cochrane Risk of Bias Tool (version 5.1.0). Data analysis was done using Stata 16.0 and Review Manager 5.4.

**Results:**

A total of 25 studies were included, covering 11 distinct auriculotherapies, and 2106 patients with PI diagnoses were included. The results of the traditional comparison showed that both auricular acupressure and transcutaneous auricular vagus nerve stimulation were more effective than the control group at lowering Pittsburgh Sleep Quality Index scores. According to the findings of the network meta-analysis, 11 auriculotherapies were more effective than sham stimulation or usual care at treating PI. According to the surface under the cumulative ranking curve values, the following are the top 5 interventions, in order of probability: Auricular bloodletting + auricular acupressure (85.8%), auricular massage + auricular scraping + auricular acupressure (85.5%), auricular scraping + auricular acupressure (84.8%), auricular needle-embedding + auricular acupressure (71.2%), and auricular acupuncture + auricular acupressure (67.5%).

**Conclusion:**

The most effective auriculotherapy regimens for PI are auricular bloodletting + auricular acupressure and auricular massage + auricular scraping + auricular acupressure. However, because of the small number of studies included in the current meta-analysis, these results need to be confirmed. Future studies should focus on high-quality, suitably powered randomized controlled trials that compare these auriculotherapy modalities head-to-head.

## 1. Introduction

Insomnia (called Bùmèi in traditional Chinese medicine [TCM]) is a common sleep problem that impairs daytime performance due to ongoing issues with sleep initiation, maintenance, or quality.^[1]^ Approximately 10% of adults are diagnosed with insomnia disorder, while an additional 20% experience occasional insomnia symptoms.^[2]^ Primary insomnia (PI) and secondary insomnia are 2 subtypes of insomnia. PI is characterized by the lack of a discernible etiology and is unaffected by comorbidities, drugs, or side effects.^[3]^ The risks of cardiovascular diseases and mood disorders (such as anxiety and depression) are markedly increased by chronic insomnia.^[4–7]^

Commonly used therapeutic approaches exhibit significant limitations: pharmacotherapy carries adverse effects,^[8]^ cognitive behavioral therapy for insomnia faces adherence challenges.^[4]^ Insomnia is attributed in TCM etiology to visceral dysfunction, nutritive-defensive *qi* discord, and *Yin-Yang* imbalance.^[9]^ According to TCM, auriculotherapy works by dredging meridians, moving *Qi-blood*, and modifying visceral *Yin-Yang* equilibrium to externally cure interior diseases.^[10]^ According to contemporary biomedical thinking, the auricular concha has a dense vagal innervation,^[11]^ and activating these pathways reduces the excitability of the hypothalamic-pituitary-adrenal axis,^[12]^ lessens negative feelings, and eases the body’s natural transition to sleep.^[13]^

Randomized controlled trials (RCTs) evaluating auriculotherapy for PI have accumulated globally,^[14]^ demonstrating significant improvements in sleep outcomes.^[15–18]^ According to the guidelines, auriculotherapy is recommended for insomnia, and this therapy has no known negative effects.^[4]^ Multiple auriculotherapy modalities exist – including auricular acupressure (Fig. S1A, Supplemental Digital Content, https://links.lww.com/MD/R719), auricular bloodletting (Fig. S1B, Supplemental Digital Content, https://links.lww.com/MD/R719), auricular needle-embedding (Fig. S1C, Supplemental Digital Content, https://links.lww.com/MD/R719), auricular acupuncture (Fig. S1D, Supplemental Digital Content, https://links.lww.com/MD/R719), auricular scraping (Fig. S1E, Supplemental Digital Content, https://links.lww.com/MD/R719), auricular massage (Fig. S1F, Supplemental Digital Content, https://links.lww.com/MD/R719), and transcutaneous auricular vagus nerve stimulation (taVNS) – which can be administered either individually or in combination. However, no evidence syntheses exist to determine the optimal technique for improving sleep quality parameters, nor have clinical practice guidelines addressed this evidence gap.

Therefore, this study employs a network meta-analysis (NMA) to evaluate and rank the efficacy of various auriculotherapy modalities for insomnia, aiming to provide evidence-based guidance for clinical decision-making.

## 2. Methods

This study was prospectively registered with the International Prospective Register of Systematic Reviews (PROSPERO; ID: CRD420251038049, https://www.crd.york.ac.uk/PROSPERO/view/CRD420251038049) and adheres to the Preferred Reporting Items for Systematic Reviews and Meta-Analyses (PRISMA) Extension Statement reporting guidelines.^[19]^

### 2.1. Inclusion and exclusion criteria

The study population comprised insomnia patients aged ≥18 years meeting diagnostic criteria specified in the Chinese Classification of Mental Disorders-3, International Classification of Sleep Disorders-3, Chinese Guidelines for the Diagnosis and Treatment of Insomnia, and the Diagnostic and Statistical Manual of Mental Disorders. Interventions included the observation group, which received either single or combined auriculotherapy, compared against a control group that received different auriculotherapies, oral estazolam, sham stimulation, or usual care. Multi-arm trials were required to contain at least 2 arms satisfying these criteria. Sham stimulation was rigorously defined as: auricular taping using adhesive patches devoid of therapeutic media (*Vaccaria segetalis* seeds or magnetic beads) or applied at nontherapeutic acupoints for insomnia, or taVNS delivered at non-vagal innervation sites or via zero-current devices. Usual care was restricted to health education without adjunctive therapies. Primary and secondary outcomes were the Pittsburgh Sleep Quality Index scores and safety assessments, respectively. Only RCTs published in Chinese or English were eligible for inclusion. Exclusion criteria included unavailable full-text articles, duplicate publications, and studies rated as grade C (indicating high risk of bias) during quality assessment.

### 2.2. Search strategy

Eight electronic databases – China National Knowledge Infrastructure, Wanfang Database, SinoMed, VIP Database (Chongqing VIP Information Co., Ltd.), PubMed, Web of Science, Embase, and Cochrane Library – were systematically searched from inception to July 28, 2025 (Fig. S2, Supplemental Digital Content, https://links.lww.com/MD/R719, for the search results of each database: Figure S2A [China National Knowledge Infrastructure], Supplemental Digital Content, https://links.lww.com/MD/R719; Fig. S2B [Wanfang Database], Supplemental Digital Content, https://links.lww.com/MD/R719; Fig. S2C [SinoMed], Supplemental Digital Content, https://links.lww.com/MD/R719; Fig. S2D [VIP Database], Supplemental Digital Content, https://links.lww.com/MD/R719; Fig. S2E [PubMed], Supplemental Digital Content, https://links.lww.com/MD/R719; Fig. S2F [Web of Science], Supplemental Digital Content, https://links.lww.com/MD/R719; Fig. S2G [Embase], Supplemental Digital Content, https://links.lww.com/MD/R719; and Fig. S2H [Cochrane Library], Supplemental Digital Content, https://links.lww.com/MD/R719). The search strategy employed a combination of subject headings and free-text terms. Search terms comprised: auriculotherap*, ear acupuncture, ear acupressure, auricular needling, auricular acupuncture, ear scraping, ear acupoint bloodletting, auricular moxibustion, auricular vagus nerve, the vagus nerve of the concha, transcutaneous vagus nerve stimulation, taVNS, sleep initiation and maintenance disorders, DIMS, disorders of initiating and maintaining sleep, sleeplessness, insomnia disorder*, insomnia*, randomized controlled trial, controlled clinical trial, randomized. To find further material, manual searches of reference lists were also conducted (Table S1, Supplemental Digital Content, https://links.lww.com/MD/R720, for the search strategies of each database).

### 2.3. Study selection and data extraction

Retrieved records were imported into NoteExpress software (Beijing Aegean Sea Music Technology Co., Ltd., Beijing, China) for duplicate removal, combining automated deduplication with manual verification to ensure unique citations. Preliminary screening was conducted by reviewing titles and abstracts against predefined inclusion and exclusion criteria. Subsequently, a full-text assessment of potentially eligible articles was performed to determine final inclusion. The entire screening process was independently executed by 2 trained researchers with cross-verification to ensure reliability and accuracy. Discrepancies were resolved through consultation with a third arbitrator. Extracted data encompassed: publication title and authorship, year of publication, participant characteristics, intervention, auricular laterality (unilateral/bilateral stimulation), primary acupoints, and outcome metrics.

### 2.4. Risk of bias assessment

Methodological quality was independently assessed by 2 trained investigators using the Cochrane Risk of Bias Tool (version 5.1.0; which was developed by The Cochrane Collaboration), with discrepancies resolved through third-party adjudication. Assessments encompassed 7 domains specified in the Cochrane Handbook for Systematic Reviews of Interventions: random sequence generation, allocation concealment, blinding of participants and personnel, blinding of outcome assessment, incomplete outcome data, selective outcome reporting, and other sources of bias. “Low risk,” “High risk,” and “Unclear risk” were assigned to each domain; studies were subsequently classified into 3 tiers: grade A (low risk across all domains), grade B (low risk in ≥ 1 domain but not all), and grade C (high/unclear risk in all domains), with grade C studies excluded from quantitative synthesis.

### 2.5. Statistical methods

Review Manager 5.4 (which was developed by The Cochrane Collaboration) was used for quality assessment and traditional pairwise meta-analysis. In Stata 16.0 (StataCorp LLC, College Station), NMA was performed. For networks containing closed loops, global and local inconsistency tests were initially applied; consistency models were used for data analysis when the inconsistency factor *P* > .5. Mean differences (MDs) with 95% confidence intervals (CIs) were used as effect size measurements because all outcome measures were continuous variables evaluated using the same tools. Treatment rankings were generated by calculating the surface under the cumulative ranking curve (SUCRA); higher SUCRA values (which ranged from 0%–100%) suggested a higher likelihood that an intervention would be the best course of action. Publication bias was evaluated using funnel plots constructed in Stata 16.0.

## 3. Results

### 3.1. Search results

**Figure 1.**
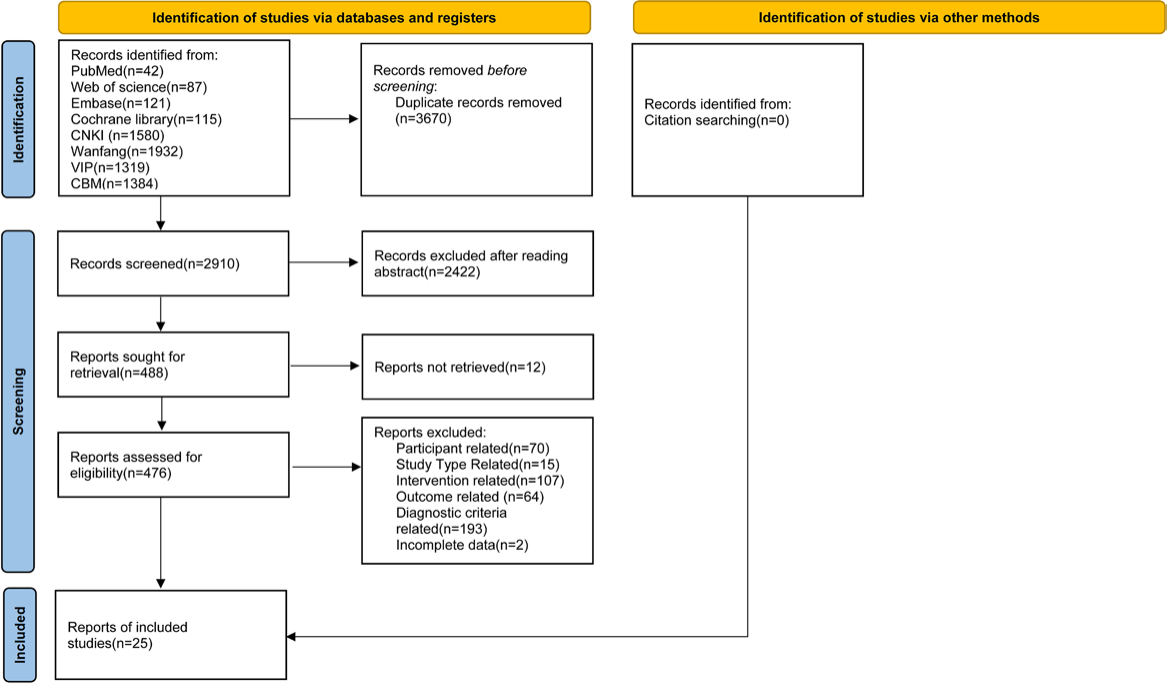
Flowchart of literature screening. CNKI = China National Knowledge Infrastructure.

A total of 6580 records were initially identified. After removing 3444 duplicates through automated deduplication in NoteExpress and an additional 226 duplicates via manual verification, 2910 unique citations remained. Title and abstract screening excluded 2422 records, leaving 488 potentially eligible articles. Full-text assessment of these articles resulted in the final inclusion of 25 RCTs.^[20–44]^ The PRISMA flow diagram detailing this process is presented in Figure 1.

### 3.2. Characteristics and methodological quality of included studies

**Figure 2.**
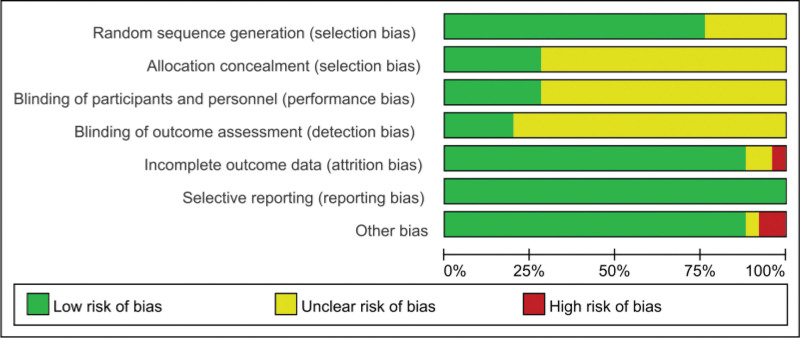
Bias risk assessment of the included studies.

The 25 included studies comprised 20 Chinese-language and 5 English-language publications, of which 24 were conducted in China and 1 in Korea. Study designs included: 1 4-arm RCT (3 arms were eligible), 2 3-arm RCTs (1 with 2 eligible arms, another with all 3 arms were eligible), and 22 2-arm RCTs, collectively involving 2106 participants and evaluating 11 auriculotherapy modalities (5 monotherapies; 6 combination regimens), as detailed in Table 1. Methodological quality assessment yielded 3 grade A studies (low risk across all bias domains) and 22 grade B studies. Random sequence generation methods were explicitly reported in 19 studies, whereas 6 studies only mentioned “randomization” without methodological specifications. Allocation concealment using opaque sealed envelopes was implemented in 7 studies, and blinding procedures were applied in 7 studies (Fig. 2 for bias risk assessment of the included studies; Fig. S3, Supplemental Digital Content, https://links.lww.com/MD/R719 for risk of bias summary about each risk of bias item for each included study; Table S2, Supplemental Digital Content, https://links.lww.com/MD/R721, for methodological quality of included studies).

**Table 1:** Baseline characteristics of the included studies.

Study	Sample size		Intervention		One or two ears	Intervention period	Intervention frequency	Follow-up	Principal acupoints	Outcome
	Intervention	Control	Intervention	Control						
Lin 2007^[20]^	30	30	Auricular acupressure	Sham stimulation	1 ear	45 d	Every 3 d	No follow-up	Shenmen, heart, and endocrine	①
Li 2010^[22]^	35	28	Auricular bloodletting + auricular acupressure	Auricular acupressure	1 ear	24 d	Every 2 d	No follow-up	Shenmen, liver, spleen, brain point, occiput, and endocrine	①
Jiang et al^[21]^	63	62	Auricular acupressure	Sham stimulation	2 ears	4 wk	Every 4 d	No follow-up	Shenmen, liver, heart, subcortex, occiput, kidney, neurasthenia area, and neurasthenia point	①
Pi et al^[23]^	132	121	Auricular acupressure	Sham stimulation	1 ear	8 wk	Twice a week	No follow-up	Shenmen, heart, and subcortex	①
Liang 2017^[24]^	35	35	Auricular needle-embedding	Estazolam	1 ear	4 wk	Every 2 d	No follow-up	Shenmen, sympathetic, subcortex, heart, spleen, kidney, and anterior ear lobe	①
Lin et al^[25]^	46	44	Auricular acupressure	Estazolam	1 ear	4 wk	Every 3 d	No follow-up	Shenmen, heart, subcortex, occiput, and cervical vertebrae	①
Suen et al^[26]^	51	50/46	Auricular acupressure + laser auriculotherapy	Auricular acupressure/laser auriculotherapy	1 ear	6 wk	Every 2 d	6 wk, 3, and 6 mo	Shenmen, heart, liver, spleen, kidney, occiput, and subcortex	①②
Zhou 2019^[27]^	31	32	Auricular acupuncture + auricular acupressure	Auricular acupressure	1 ear	4 wk	Twice a week	No follow-up	Shenmen, heart, occiput, subcortex, stomach, spleen, lung, triple energizer, and gallbladder	①
Chen 2021^[28]^	36	37	Auricular bloodletting + auricular acupressure	Auricular acupressure	1 ear	4 wk	Twice a week	3 mo	Shenmen, sympathetic, subcortex, ear apex, back-of-ear heart	①②
Ye 2022^[30]^	30	30	Auricular bloodletting + auricular acupressure	Auricular acupressure	1 ear	4 wk	Once a week	No follow-up	Shenmen, liver, spleen, sympathetic, subcortex, and heart	①②
Zeng et al^[31]^	32	32/31	Auricular scraping + auricular acupressure	Auricular scraping/auricular acupressure	1 ear	4 wk	Every 3 d	No follow-up	Shenmen, liver, heart, spleen, kidney, occiput, anterior ear lobe, and subcortex	①
Zhang et al^[32]^	40	40	Auricular acupressure	Usual care	–	4 wk	Twice a day	6 wk	Shenmen, heart, kidney, and insomnia point	①
Wu et al^[29]^	15	15	taVNS	Sham stimulation	1 ear	4 wk	Twice a day	No follow-up	Auricular concha area	①
Leng et al^[34]^	30	30	Auricular scraping	Estazolam	–	8 wk	Once a week	No follow-up	Shenmen, liver, heart, subcortex, occiput, and gallbladder	①
Huang et al^[35]^	48	48	Auricular scraping + auricular acupressure	Auricular acupressure	–	4 wk	Twice a week	No follow-up	Heart, endocrine, brainstem, triple energizer, anterior ear lobe, lung, kidney, and back-of-ear heart	①
Deng et al^[33]^	31	31	Auricular massage + auricular scraping + auricular acupressure	Auricular acupressure	1 ear	4 wk	Every 3–4 d	No follow-up	Shenmen, subcortex, heart, occiput, spleen, stomach, body-mind acupoints, and hypnotic point	①
Wu et al^[36]^	30	30	taVNS	Auricular acupressure	1 ear	4 wk	Twice a day	No follow-up	Auricular concha area/shenmen, heart, subcortex, and sympathetic	①
Zheng 2023^[37]^	33	34	Auricular needle-embedding + auricular acupressure	Auricular acupressure	1 ear	4 wk	Once a week	2 wk	Shenmen, heart, Liver, pancreas & gallbladder, and subcortex	①
Gong 2023^[38]^	30	30	taVNS	Sham stimulation	1 ear	4 wk	Once a day	No follow-up	Auricular concha area	①
Jing et al^[41]^	43	41	Auricular scraping + auricular acupressure	Auricular acupressure	–	8 wk	Twice a week	No follow-up	Shenmen, heart, Sympathetic, and subcortex	①
Zhang et al^[39]^	36	36	taVNS	Sham stimulation	2 ears	8 wk	Once a day	12 wk	Heart, and kidney	①②
Gu et al^[40]^	35	35	Auricular scraping + auricular acupressure	Auricular acupressure	1 ear	4 wk	Every 3–5 d	6 wk	Shenmen, heart, subcortex, anterior ear lobe, occiput, and neurasthenia area	①
Qi et al^[42]^	34	33	taVNS	Sham stimulation	1 ear	5 wk	Twice a day	No follow-up	Auricular cymba concha	①②
Yeom et al^[43]^	20	20	taVNS	Sham stimulation	2 ears	6 wk	Once a day	No follow-up	Auricular cymba concha	①②
Huang et al^[44]^	39	38	Auricular scraping + auricular acupressure	Auricular acupressure	1 ear	4 wk	Every 3 d	No follow-up	Shenmen, heart, occiput, subcortex, and kidney	①

①: PSQI; ②: safety assessment; intervention frequency refers to the frequency of intervention in the intervention group; taVNS refers to transcutaneous auricular vagus nerve stimulation.

PSQI = Pittsburgh Sleep Quality Index.

### 3.3. Direct comparison results

Traditional meta-analysis was performed using Review Manager 5.4 for auriculotherapies versus non-auricular interventions with at least 2 direct comparisons. Significant heterogeneity was observed (*P* < .05, *I*^2^ = 86%), warranting a random-effects model. The effectiveness of auriculotherapies was statistically higher than that of non-auricular interventions (*P* < .05, *Z* = 6.31, *MD* = −3.55, 95% *CI* = −4.65 to −2.45).

**Figure 3.**
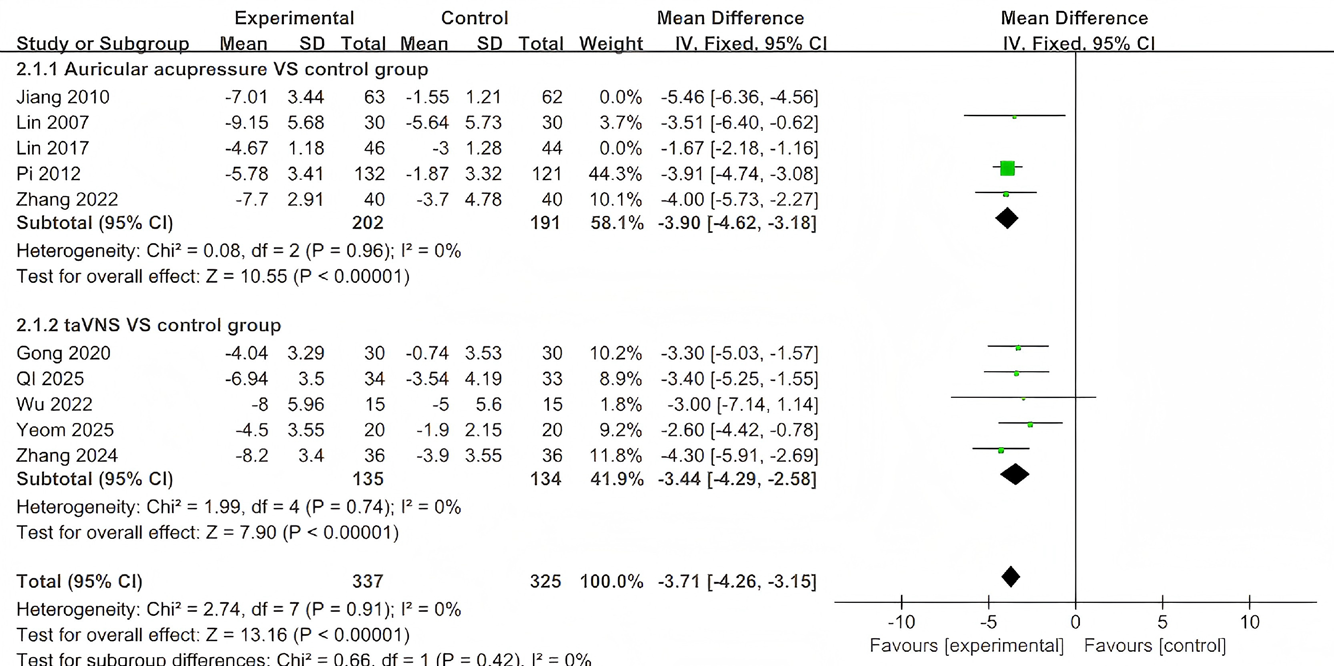
The forest plot of the effect of auriculotherapy on PSQI in patients with primary insomnia. CI = confidence interval, PSQI = Pittsburgh Sleep Quality Index, SD = standard deviation.

Two subgroups were analyzed: auricular acupressure versus control and taVNS versus control. Significant heterogeneity was identified in the auricular acupressure subgroup, according to preliminary heterogeneity testing (*P* < .05, *I*^2^ = 93%). When Jiang et al^[21]^ and Lin^[25]^ were excluded from the sensitivity analysis, there was less heterogeneity (*P* = .96, *I*^2^ = 0%), which could be attributed to Jiang’s bilateral stimulation protocol (as opposed to unilateral in other studies) and Lin’s use of an active pharmaceutical comparator (estazolam) as opposed to sham stimulation and usual care in additional trials. There was minimal heterogeneity in the taVNS subgroup (*P* = .74, *I*^2^ = 0%). Both subgroups were then subjected to fixed-effect models, which showed statistically significant decreases in Pittsburgh Sleep Quality Index scores compared to controls: auricular acupressure (*MD* = −3.90, 95% *CI* = −4.62 to −3.18; *P* < .05) and taVNS (*MD* = −3.44, 95% *CI* = −4.29 to −2.58; *P* < .05; Fig. 3).

### 3.4. NMA results

#### 3.4.1. Network evidence graph

**Figure 4.**
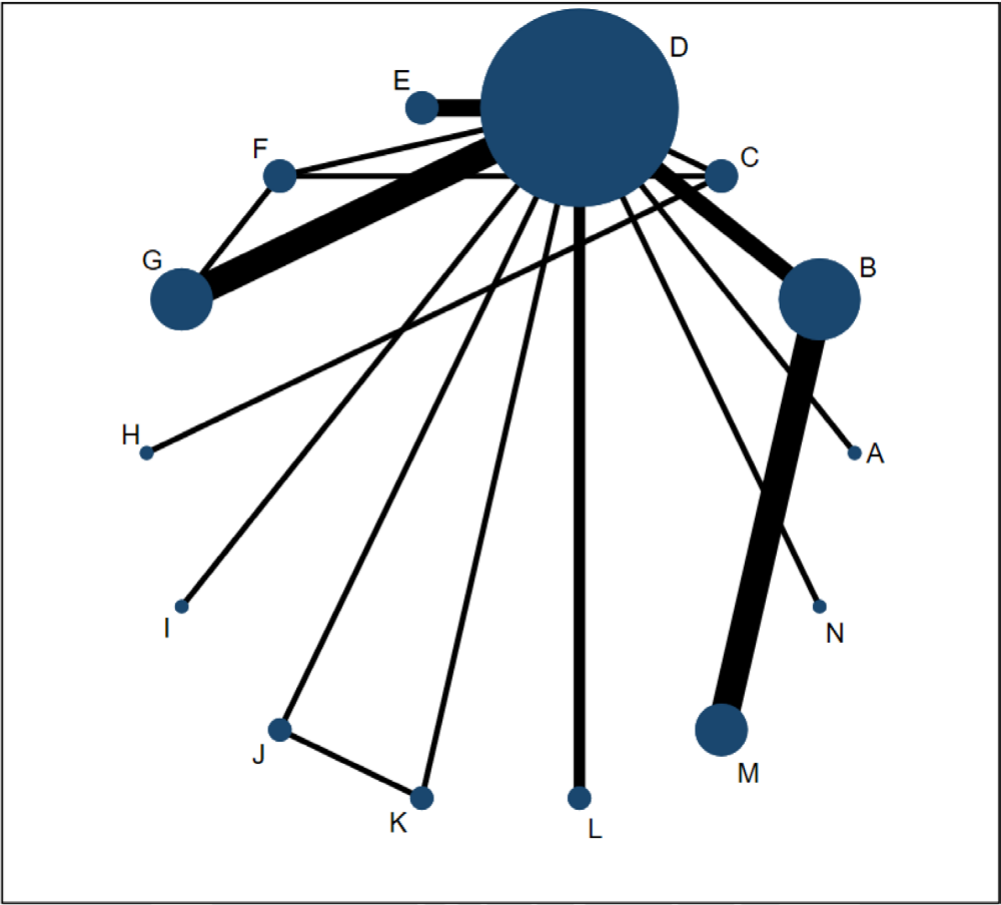
The network map for included studies. (A) Usual care; (B) sham stimulation; (C) estazolam; (D) auricular acupressure; (E) auricular bloodletting + auricular acupressure; (F) auricular scraping; (G) auricular scraping + auricular acupressure; (H) auricular needle-embedding; (I) auricular needle-embedding + auricular acupressure; (J) laser auriculotherapy; (K) auricular acupressure + laser auriculotherapy; (L) auricular massage + auricular scraping + auricular acupressure; (M) taVNS; (N) auricular acupuncture + auricular acupressure. taVNS = transcutaneous auricular vagus nerve stimulation.

The network incorporated 14 interventions forming 16 direct comparisons and 3 triangular closed loops, with node sizes proportional to intervention-specific sample sizes, connecting lines indicating direct comparisons between pairs of interventions, and line thickness corresponding to the number of comparative studies; the number of auricular acupressure and auricular scraping + auricular acupressure was more than that of other interventions for PI, with key closed loops including auricular acupressure–auricular scraping–auricular scraping + auricular acupressure and auricular acupressure–auricular scraping–estazolam (Fig. 4); the minimum dataset is shown in Table S3, Supplemental Digital Content, https://links.lww.com/MD/R721.

#### 3.4.2. Inconsistency assessment

Given the presence of closed loops, inconsistency assessment was performed where global testing indicated good overall consistency (*P* = .84 > .05; Fig. S4A, Supplemental Digital Content, https://links.lww.com/MD/R719), and subsequent node-splitting analysis confirmed local consistency across all pairwise comparisons (*P* > .05, Fig. S4B, Supplemental Digital Content, https://links.lww.com/MD/R719), thus validating the application of a consistency model for NMA.

#### 3.4.3. NMA findings

**Figure 5.**
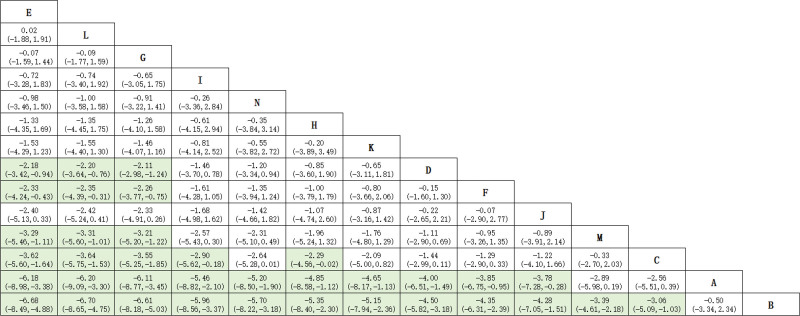
The league figure of included studies. Highlighted data indicate statistically significant differences between the comparisons. (E) Auricular bloodletting + auricular acupressure; (L) auricular massage + auricular scraping + auricular acupressure; (G) auricular scraping + auricular acupressure; (I) auricular needle-embedding + auricular acupressure; (N) auricular acupuncture + auricular acupressure; (H) auricular needle-embedding; (K) auricular acupressure + laser auriculotherapy; (D) auricular acupressure; (F) auricular scraping; (J) laser auriculotherapy; (M) taVNS; (C) estazolam; (A) usual care; (B) sham stimulation. taVNS = transcutaneous auricular vagus nerve stimulation.

The NMA showed that all 11 auriculotherapies were more effective than either sham stimulation or standard therapy for PI (Fig. 5). SUCRA rankings were: auricular bloodletting + auricular acupressure (85.8%), auricular massage + auricular scraping + auricular acupressure (85.5%), auricular scraping + auricular acupressure (84.8%), auricular needle-embedding + auricular acupressure (71.2%), auricular acupuncture + auricular acupressure (67.5%),auricular needle-embedding (61.5%), auricular acupressure + laser auriculotherapy (58.2%), auricular acupressure (44.8%), auricular scraping (42.3%), laser auriculotherapy (41.4%), taVNS (27.0%), estazolam (21.6%), usual care (5.7%), and sham stimulation (2.8%; Table 2).

**Table 2:** The SUCRA of different auriculotherapy.

Treatment	SUCRA	Rank
Auricular bloodletting + auricular acupressure	85.8	1
Auricular massage + auricular scraping + auricular acupressure	85.5	2
Auricular scraping + auricular acupressure	84.8	3
Auricular needle-embedding + auricular acupressure	71.2	4
Auricular acupuncture + auricular acupressure	67.5	5
Auricular needle-embedding	61.5	6
Auricular acupressure + laser auriculotherapy	58.2	7
Auricular acupressure	44.8	8
Auricular scraping	42.3	9
Laser auriculotherapy	41.4	10
taVNS	27	11
Estazolam	21.6	12
Usual care	5.7	13
Sham stimulation	2.8	14

SUCRA = the surface under the cumulative ranking curve, taVNS = transcutaneous auricular vagus nerve stimulation.

#### 3.4.4. Publication bias

**Figure 6.**
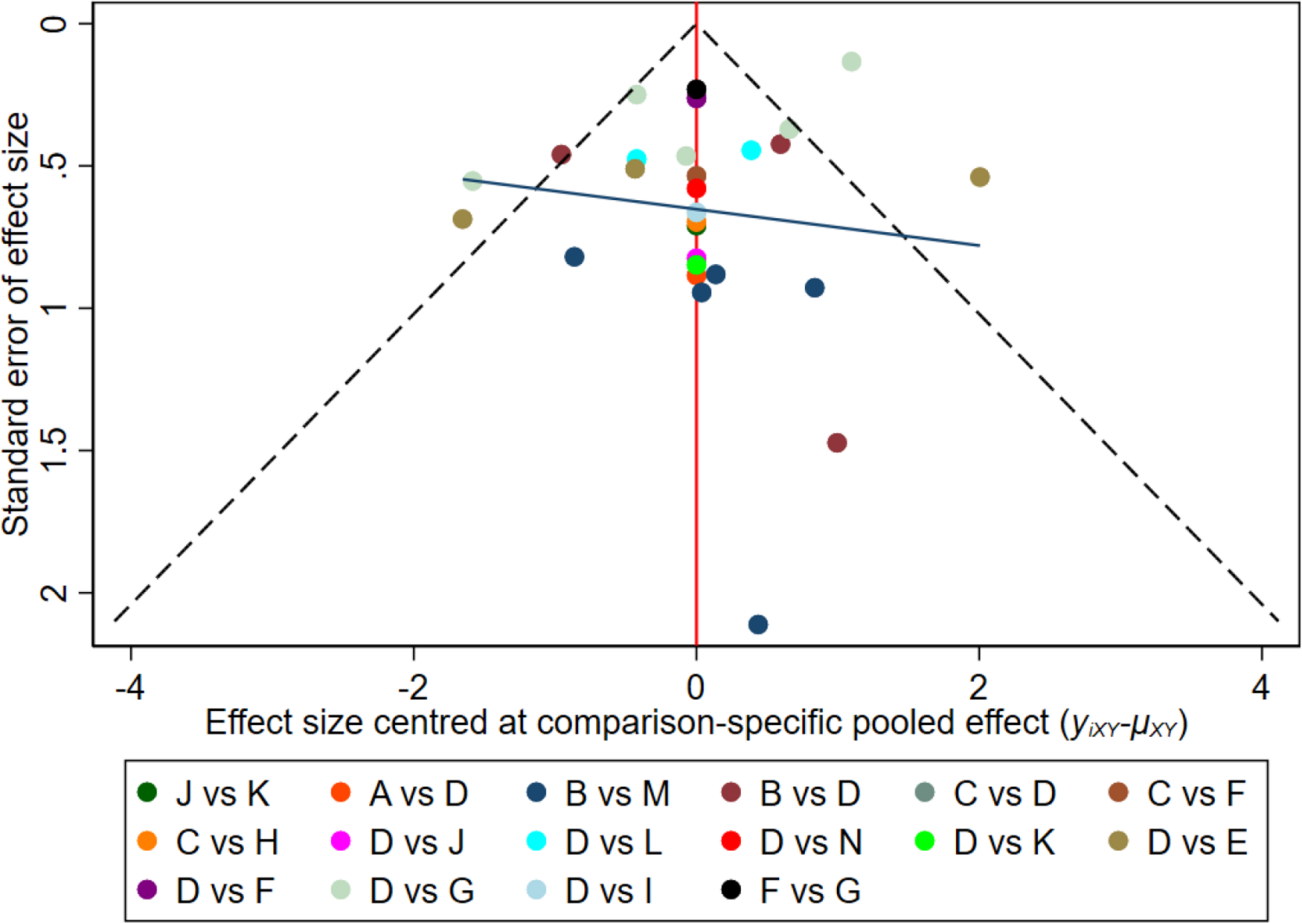
Funnel plot of included studies of PSQI. (A) Usual care; (B) sham stimulation; (C) estazolam; (D) auricular acupressure; (E) auricular bloodletting + auricular acupressure; (F) auricular scraping; (G) auricular scraping + auricular acupressure; (H) auricular needle-embedding; (I) auricular needle-embedding + auricular acupressure; (J) laser auriculotherapy; (K) auricular acupressure + laser auriculotherapy; (L) auricular massage + auricular scraping + auricular acupressure; (M) taVNS; (N) auricular acupuncture + auricular acupressure. PSQI = Pittsburgh Sleep Quality Index, taVNS = transcutaneous auricular vagus nerve stimulation.

A funnel plot was constructed for the 25 included studies. The plot demonstrated essential symmetry, with 2 studies positioned below the central symmetry axis, suggesting possible small-sample effects (Fig. 6).

### 3.5. Safety analysis

Among the 25 included studies, 6 reported safety outcomes. One study documented 16 cases of mild auricular skin irritation attributed to the adhesive tape used in auricular acupressure.^[26]^ Auricular bloodletting in 1 study resulted in a single case of auricular hematoma and 1 case of chest tightness. According to the authors’ analysis, emotional tension was the root cause of chest tightness.^[28]^ One study reported 9 adverse events related to taVNS: 7 cases of mild auricular pain due to the ear clip, 1 instance of gingival bleeding, and 1 case of dizziness.^[39]^ Another study reported 1 case of intolerable auricular pain caused by the ear clip in the sham stimulation group.^[42]^ Two studies observed no adverse events in any participants.^[30,43]^ Crucially, none of the studies identified any severe adverse outcomes.

## 4. Discussion

### 4.1. Optimal auriculotherapies for PI

NMA identified 3 optimal auriculotherapies with comparable efficacy (SUCRA difference < 1%): auricular bloodletting + auricular acupressure (85.8%), auricular massage + auricular scraping + auricular acupressure (85.5%), and auricular scraping + auricular acupressure (84.8%). These findings resonate with classical Chinese medical theory from *Lingshu • Xieke*, which posits that insomnia results from “*Yan*g failing to enter *Yin*” (*Yang Bu Ru Yin*)–a pathological state where defensive *Qi (Wei Qi*) stagnates in *Yang* meridians and cannot transition to *Yin* meridians during sleep. This commonly manifests as heart-kidney disharmony (*Xin Shen Bu Jiao*), characterized by disrupted *Yin-Yang* equilibrium where heart *Yang* fails to warm kidney water and kidney *Yin* cannot nourish the heart.^[45]^ Modern medicine attributes insomnia pathogenesis to cortical dysfunction and autonomic nervous system dysregulation.^[46]^ The superior efficacy of these combinations reflects synergistic East-West mechanisms: TCM holds that auricular stimulation balances *Yin-Yang* and regulates visceral function through organ-meridian correspondences,^[10]^ while neuroanatomical studies confirm the auricle’s rich vagal innervation enables modulation of sympathetic-parasympathetic balance and cortical excitability when stimulating acupoints (e.g., heart, kidney, subcortex).^[47–49]^ Specifically, auricular bloodletting at the ear apex (Er jian) – the primary site for auricular bloodletting in insomnia management – rapidly clears heat and irritability by modulating microcirculation, stimulating the lesser occipital nerve, and regulating hormonal and neurotransmitter levels in insomnia patients,^[50,51]^ while auricular acupressure provides sustained vagal stimulation to maintain autonomic equilibrium.^[47,48]^ Their integration forms an acute-chronic synergistic model superior to monotherapy, a finding consistent with previous research.^[52]^ Similarly, the combined auricular scraping and auricular acupressure protocol involves first gently scraping specific auricular acupoints along meridian pathways to dredge collaterals and mobilize *Qi* and blood,^[53]^ followed by auricular acupressure at corresponding sites. This sequential approach integrates the “*Qi*” mobilized during scraping with sustained acupressure stimulation, enhancing meridian conduction and regulating visceral *Yin-Yang* balance to improve sleep quality. However, the original studies indicate that preparatory auricular massage is required to optimize *Qi*-blood flow and therapeutic efficacy.^[31,34,41]^ Consequently, the combined auricular scraping and auricular acupressure protocol demonstrates operational equivalence to triple auriculotherapy (auricular massage + auricular scraping + auricular acupressure). Therefore, current evidence supports the prioritization of auricular scraping + auricular acupressure or triple auriculotherapy as a preferred treatment option for PI, consistent with previous research.^[54]^ The treatment duration for these 2 modalities was typically 4 weeks, with a few studies adopting 8 weeks, and the treatment frequency was predominantly twice a week, with a minority of studies using once a week. Given the limited number of included studies and the insufficient methodological rigor of some included literature, the difference in efficacy between the 2 approaches requires validation through more large-sample, high-quality studies. Furthermore, additional comparative research is warranted to optimize their treatment duration and frequency.

### 4.2. Enhanced efficacy of multimodal auriculotherapy for insomnia

SUCRA rankings demonstrate that high-efficacy interventions include auricular bloodletting + auricular acupressure, auricular massage + auricular scraping + auricular acupressure, auricular scraping + auricular acupressure, auricular needle-embedding + auricular acupressure, and auricular acupuncture + auricular acupressure – all classified as multimodal auriculotherapies. In contrast, single-modality approaches (auricular acupressure, auricular scraping, laser auriculotherapy, and taVNS) ranked lower, confirming the superior efficacy of combined therapies. Mechanistically, auricular needle-embedding enhances stimulation intensity and prolongs therapeutic duration when combined with auricular acupressure through acute-phase neuromodulation and sustained effects,^[37]^ while auricular acupuncture provides immediate high-intensity stimulation (“impact stimulation”), synergizing with auricular acupressure’s maintenance stimulation for amplified therapeutic response.^[27]^ Consequently, clinicians should implement syndrome differentiation (pattern differentiation) to develop personalized comprehensive auriculotherapy regimens based on patients’ biopsychosocial profiles, socioeconomic factors, as well as intervention feasibility and accessibility, thereby optimizing therapeutic outcomes.

### 4.3. Favorable safety profile of auriculotherapy for PI

Among the 6 studies reporting safety data within this NMA, adverse events were exclusively mild and included skin irritation from auricular acupressure adhesives, clip-induced discomfort during taVNS, and localized hematomas following auricular bloodletting, collectively indicating a favorable safety profile for auriculotherapy. However, the limited number of safety reports covering only 3 intervention modalities, combined with inadequate safety outcome metrics, necessitates future high-quality clinical studies specifically designed to establish more reliable evidence regarding auriculotherapy safety in PI management.

### 4.4. Strengths and limitations of this study

This study employed a combined approach of traditional meta-analysis and NMA to evaluate the efficacy of auriculotherapy in patients with PI. Although a previous meta-analysis has confirmed the favorable therapeutic effect of auriculotherapy for PI,^[16]^ several clinical dilemmas persist in practice: Should multiple auriculotherapies be combined or used individually? Should clinicians choose short-term, high-intensity stimulation (e.g., auricular acupuncture) or interventions with sustained effects (e.g., auricular acupressure)? What approach can both conserve medical resources and ensure optimal patient outcomes? Moreover, no corresponding guidelines or comprehensive evidence synthesis articles were identified to address these questions. The present study first demonstrated the overall favorable efficacy of auriculotherapies through traditional meta-analysis, and then applied NMA to compare the differential effectiveness among various auricular interventions, identifying the most likely optimal therapy. These findings offer evidence-based guidance for clinical decision-making regarding auriculotherapy selection.

Notable limitations warrant rigorous consideration: methodological deficiencies in original studies – including unreported randomization methods, unblinded designs, and heterogeneity in acupoint selection, treatment duration, and stimulation intensity – may introduce bias. Furthermore, some interventions were evaluated in single studies, compromising statistical reliability. To address these constraints, future RCTs should strictly adhere to CONSORT and STRICTA reporting guidelines to enhance methodological quality, while multicenter head-to-head trials with larger samples are warranted to definitively establish comparative efficacy.

## 5. Conclusion

Accumulating evidence demonstrates significant therapeutic efficacy of auriculotherapy for insomnia management. This NMA confirms that all 11 evaluated auriculotherapies exhibit clinically meaningful benefits in PI. We recommend auricular bloodletting + auricular acupressure and auricular massage + auricular scraping + auricular acupressure as the first choice of auriculotherapy treatment for improving PI. Furthermore, to optimize therapeutic efficacy, clinicians should implement personalized comprehensive auriculotherapy protocols – tailored to individual symptom profiles, healthcare resource constraints, and patient preferences – rather than applying isolated single-modality interventions.

## Author contributions

**Conceptualization:** Zhaolong Luo.

**Funding acquisition:** Zhaolong Luo.

**Data curation:** Jiao Shen, Qiong Zhang, Jiangyan Xiong.

**Formal analysis:** Jiao Shen.

**Writing – original draft:** Zhaolong Luo, Jiao Shen.

**Writing – review & editing:** Xiangqin Chen, Jiangyan Xiong.

## Supplementary Material






