# Supplementary material for: Efficacy of auriculotherapies for primary insomnia: A systematic review and network meta-analysis
Source: Medicine (Baltimore). 2026 Apr 24;105(17):e48357. doi: 10.1097/MD.0000000000048357 (PMC13124389; doi:10.1097/MD.0000000000048357)
Supplement: Supplementary file 1 [file medi-105-e48357-s001.pdf]

Figure S1. The brief introduction of auriculotherapies: (A) Auricular acupressure; (B) Auricular bloodletting; (C) Auricular needle-embedding; (D) Auricular acupuncture; (E) Auricular scraping; (F) Auricular massage.

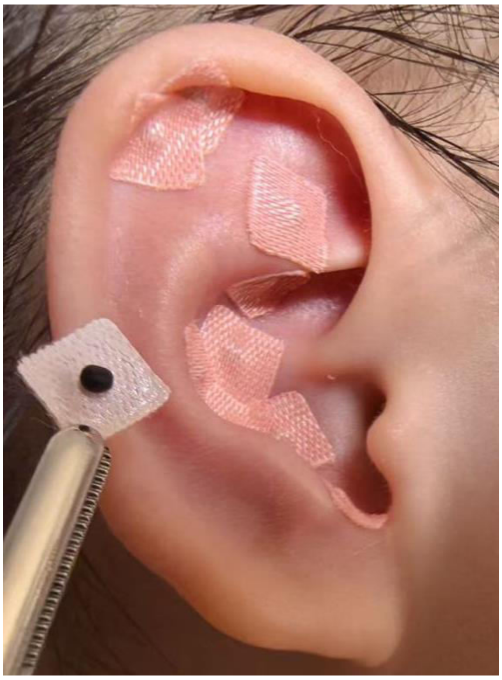

A: Auricular acupressure: Auricular acupressure involves taping organic seeds, such as green beans, rice, and Vaccaria, or magnetic pellets on acupoints located on the external ears, and then pressing down on the acupoints with a gentle force until the sensation of burning, soreness, numbness, distension, or heat is felt.

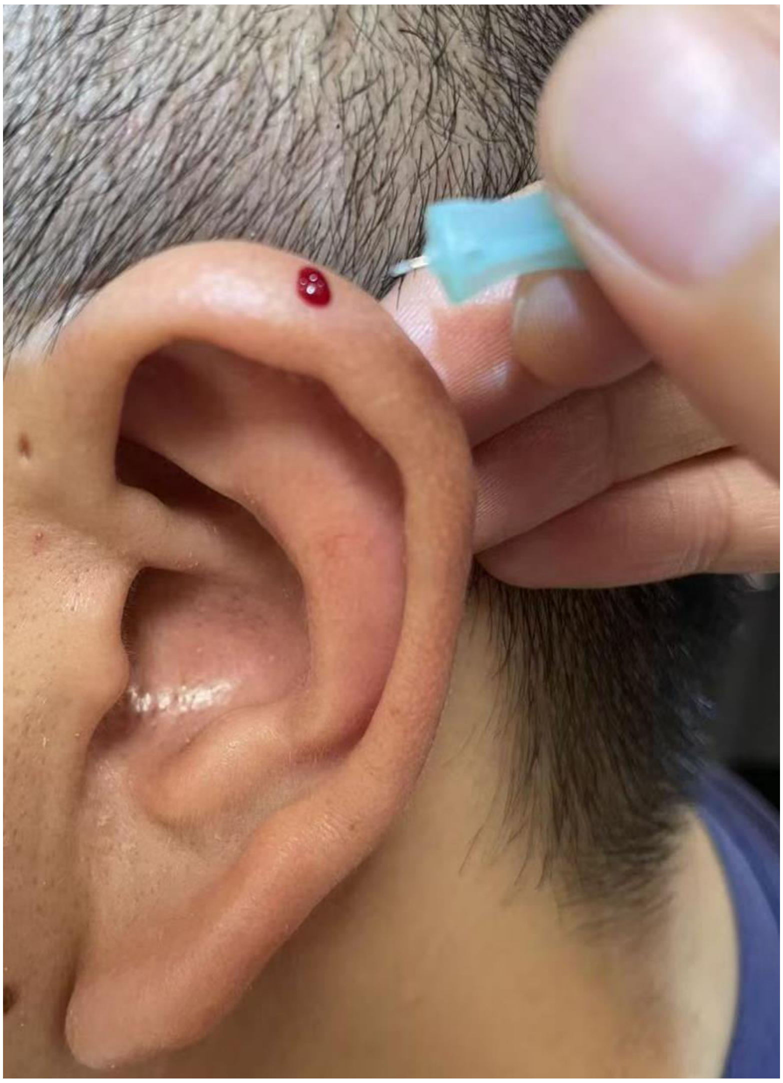

B: Auricular bloodletting: Auricular bloodletting is a therapeutic method that involves using specific instruments to perform needling, pricking, or scarifying on auricular points or blood vessels of the ear auricle to release blood, for the purpose of preventing and treating diseases.

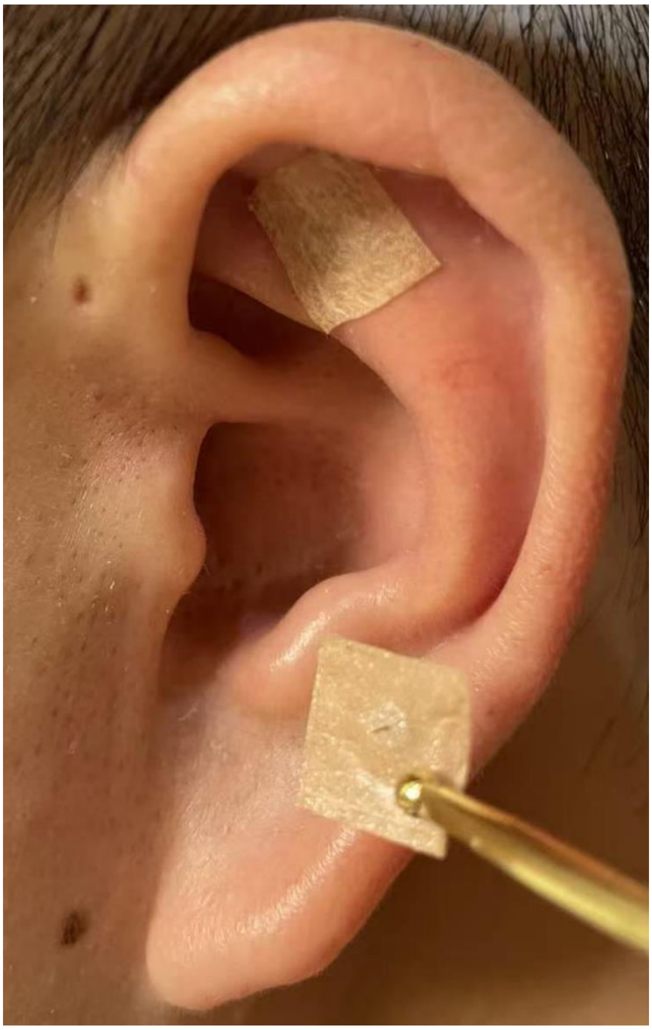

C: Auricular needle-embedding: Auricular needle-embedding is a method that involves the insertion and fixation of an intradermal needle (a special type of small needle inserted into and retained at the auricular point) to provide sustained stimulation for the purpose of preventing and treating diseases.

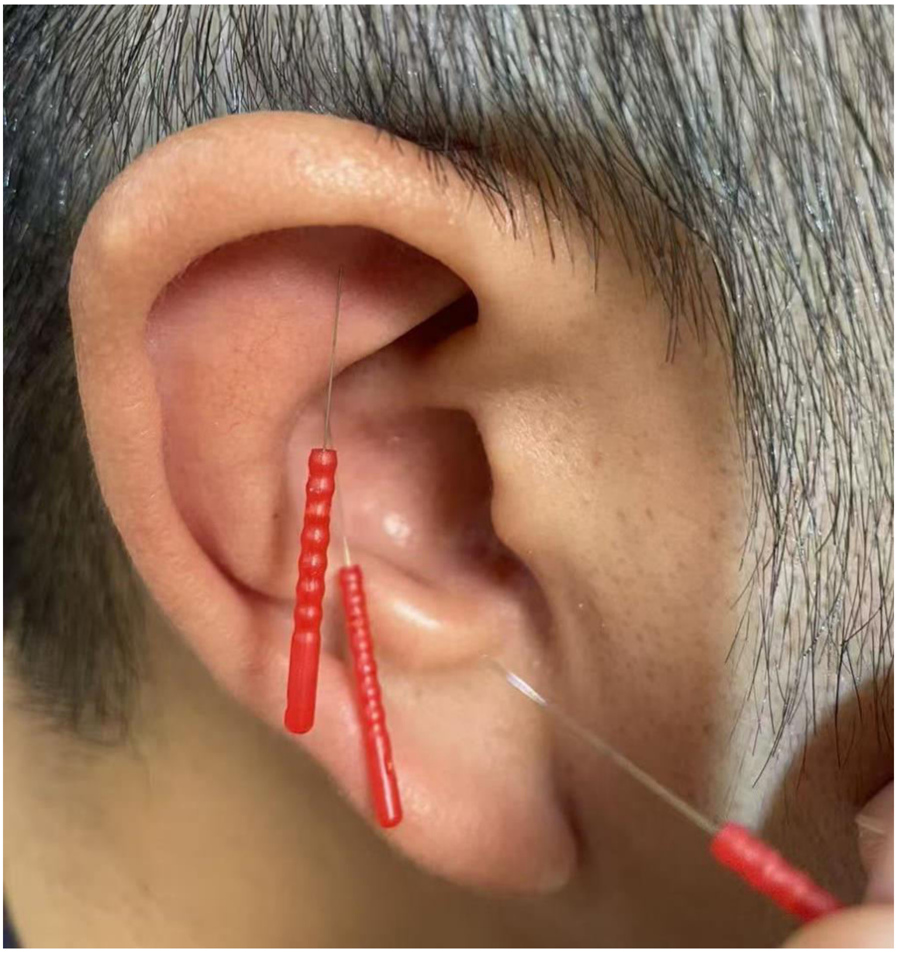

D: Auricular acupuncture: Auricular acupuncture is a method that involves stimulating auricular points with filiform needles (the fine needles typically used in acupuncture) to prevent and treat diseases.

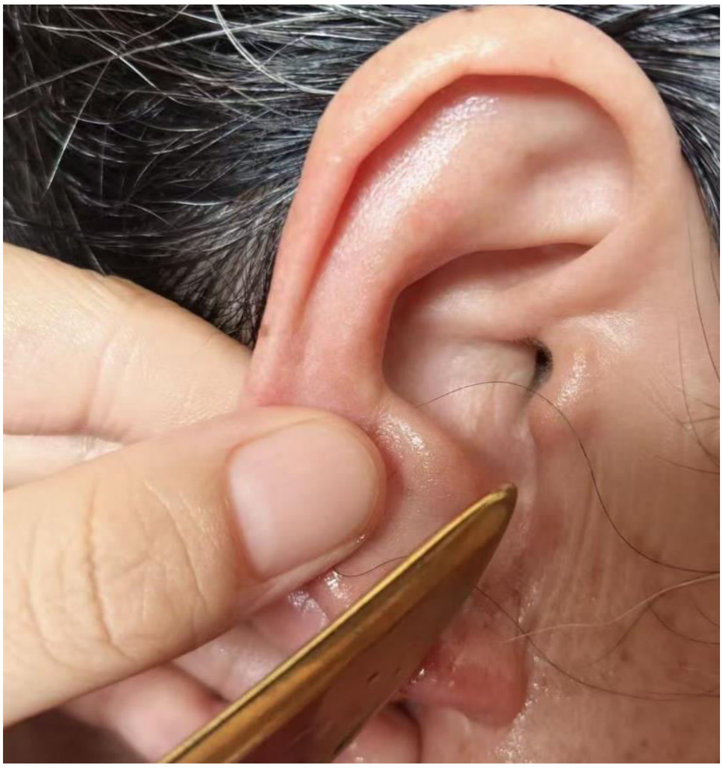

E: Auricular scraping: Auricular scraping is a technique that involves applying gentle scraping motions with a specific tool to stimulate specific auricular points, thereby achieving the purpose of treating and preventing diseases.

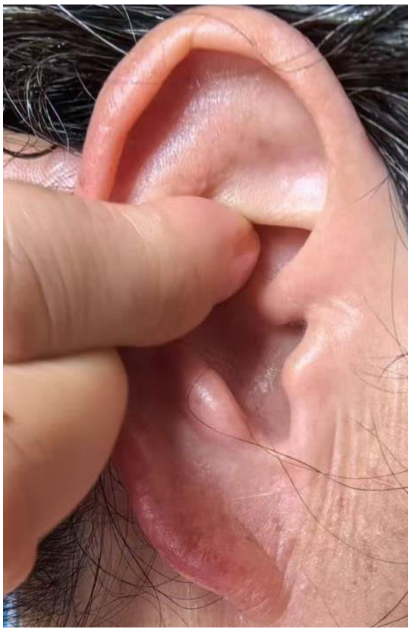

F: Auricular massage: Auricular massage is a method in which either a practitioner or the patient themselves uses the fingertip to apply massage techniques such as pressing, rubbing, pinching, and lifting to specific areas of the auricle. These manipulations induce distinct local sensations, including soreness, numbness, pain, distension, and warmth, with the purpose of preventing and treating diseases as well as promoting health maintenance.

Figure S2. Results of Literature Search in Various Databases: (A)CNKI; (B)Wanfang Database; (C) VIP Database ; (D) Sinomed; (E) PubMed; (F) Web of science; (G) Embase; (H) Cochrane Library.

A. China National Knowledge Infrastructure (CNKI): 1580

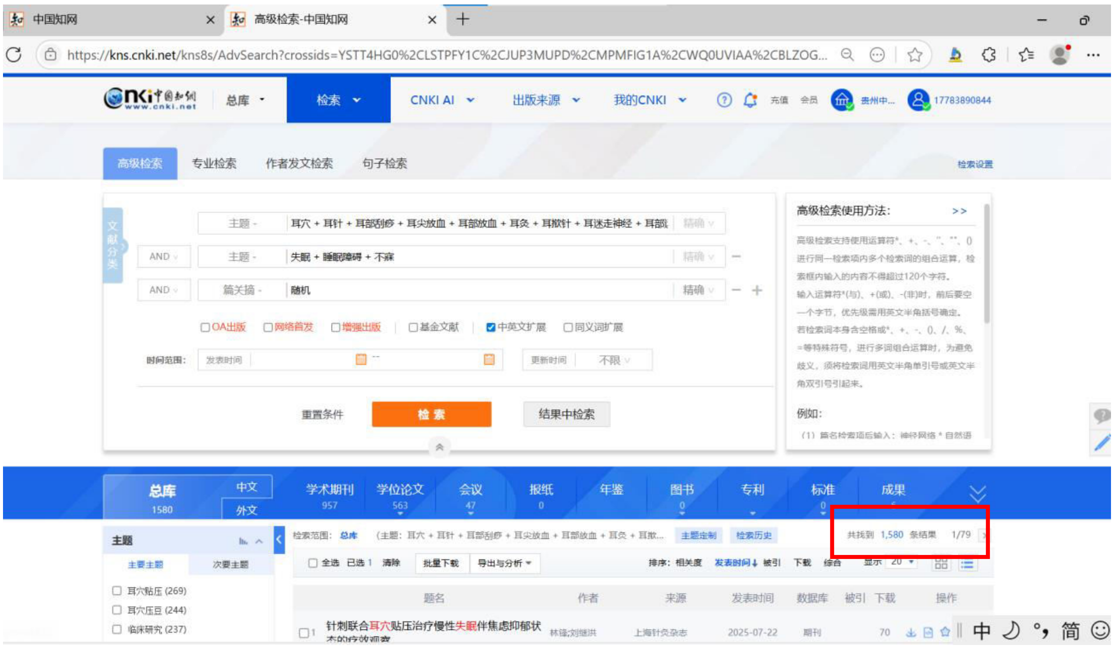

B. Wanfang Database：1932

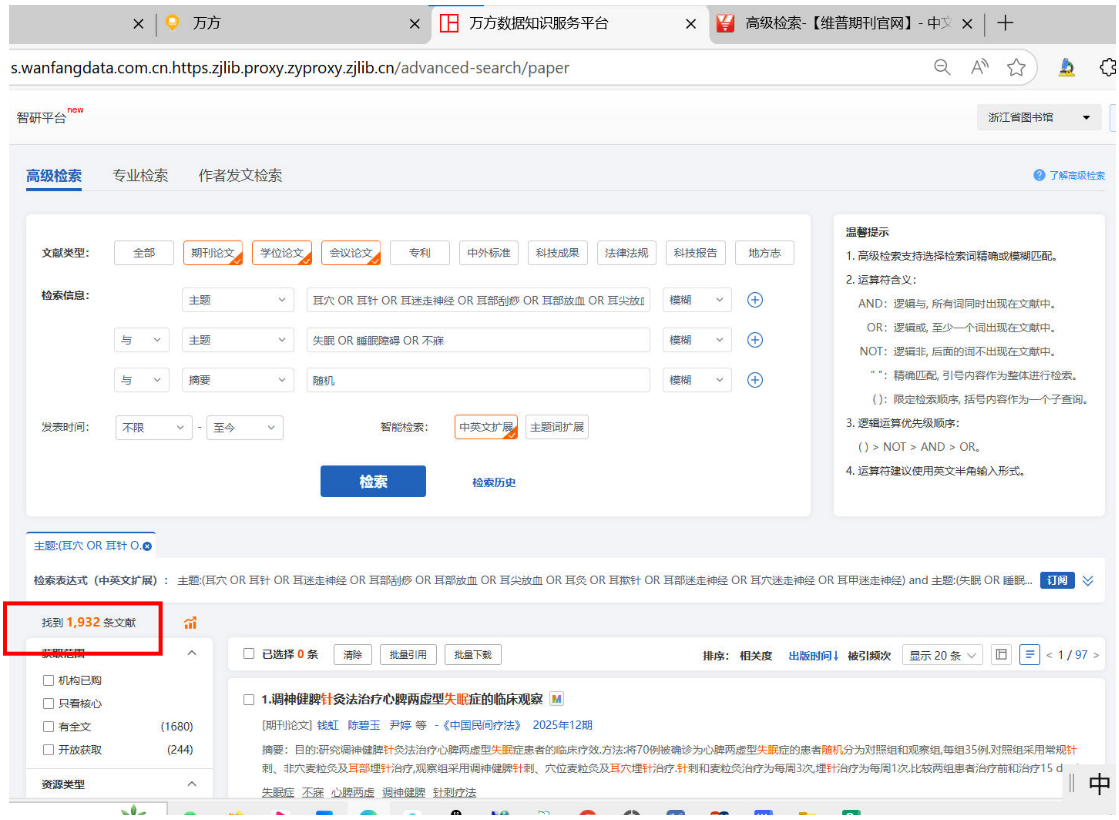

C. VIP Database: 1319

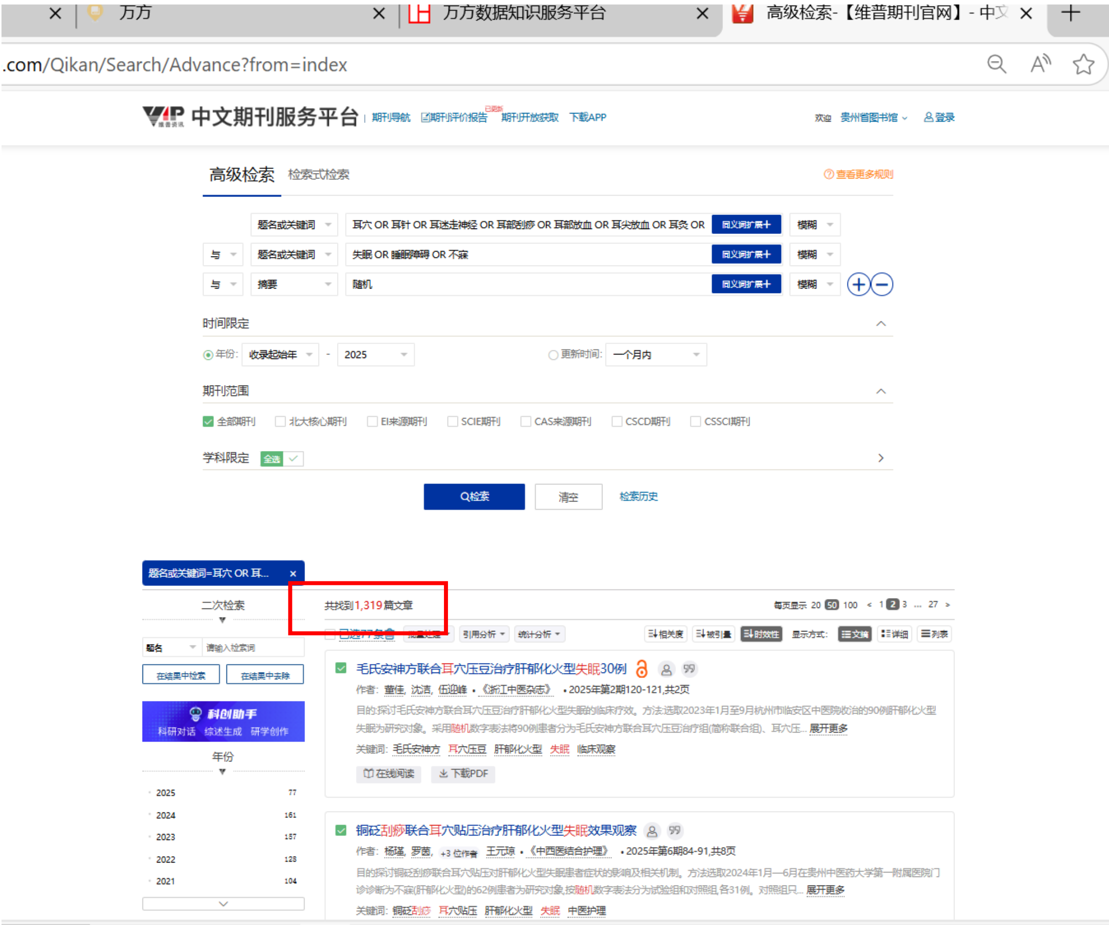

D. Sinomed: 1384

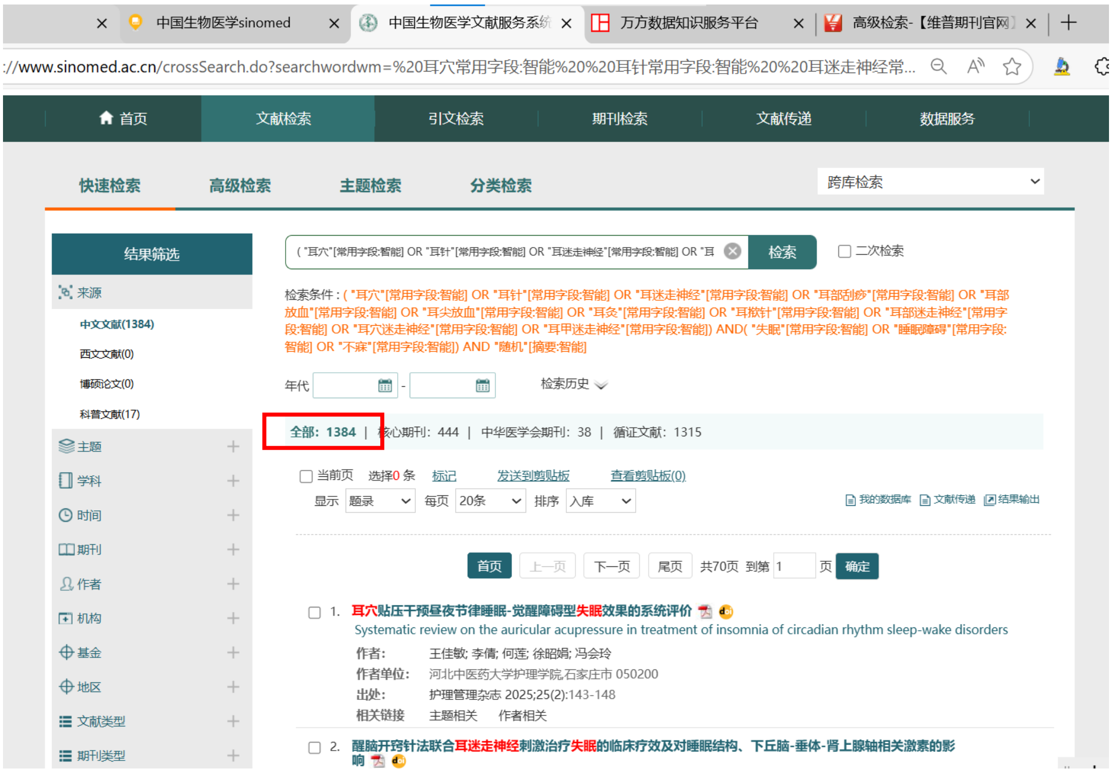

E. PubMed: 42

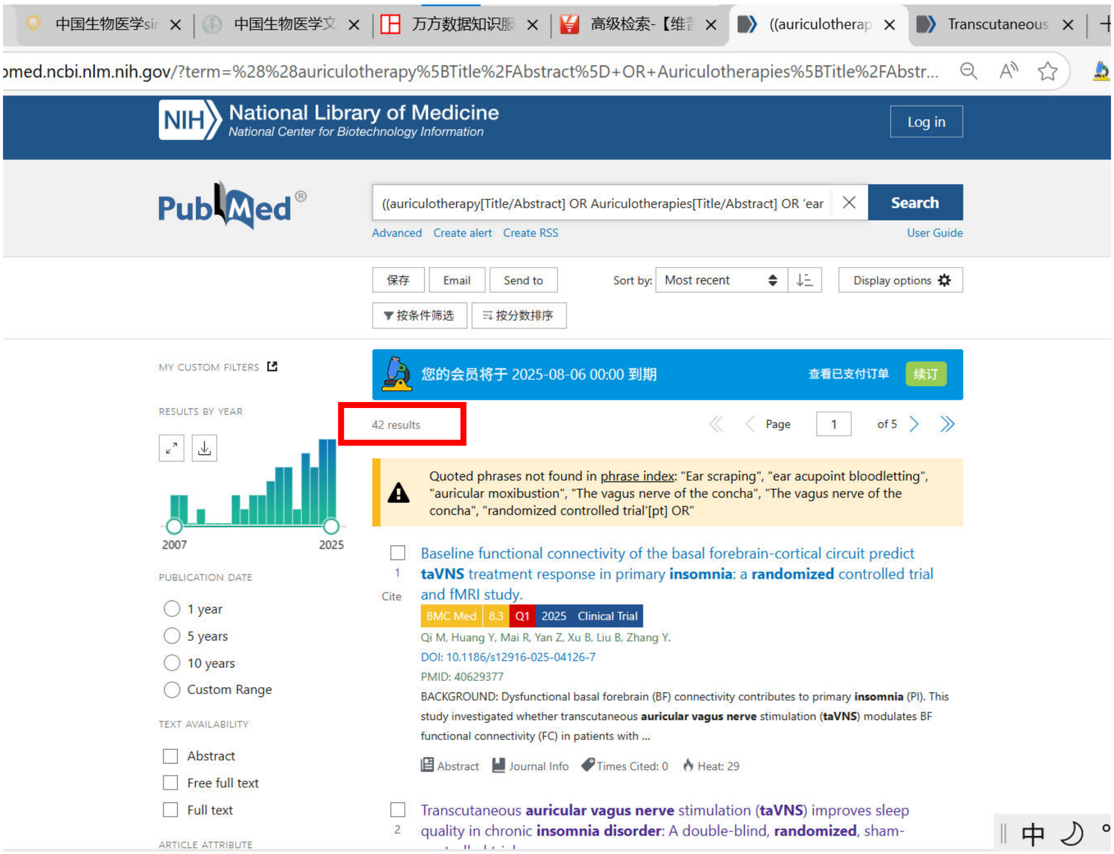

F. Web of science: 87

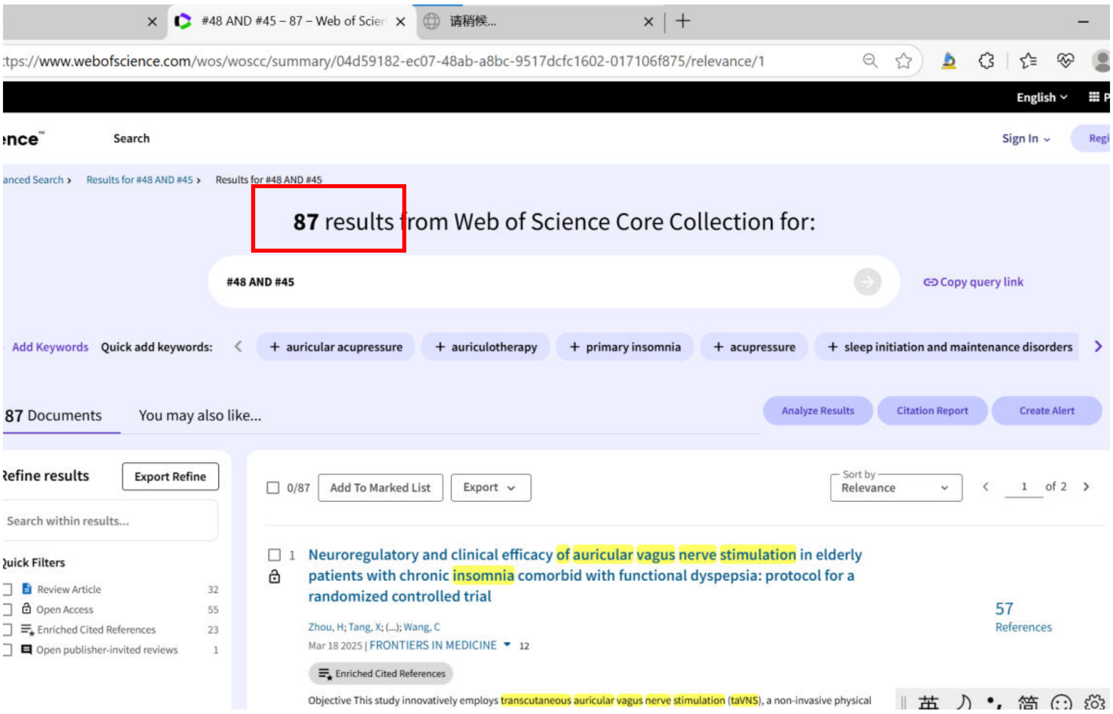

G. Embase: 121

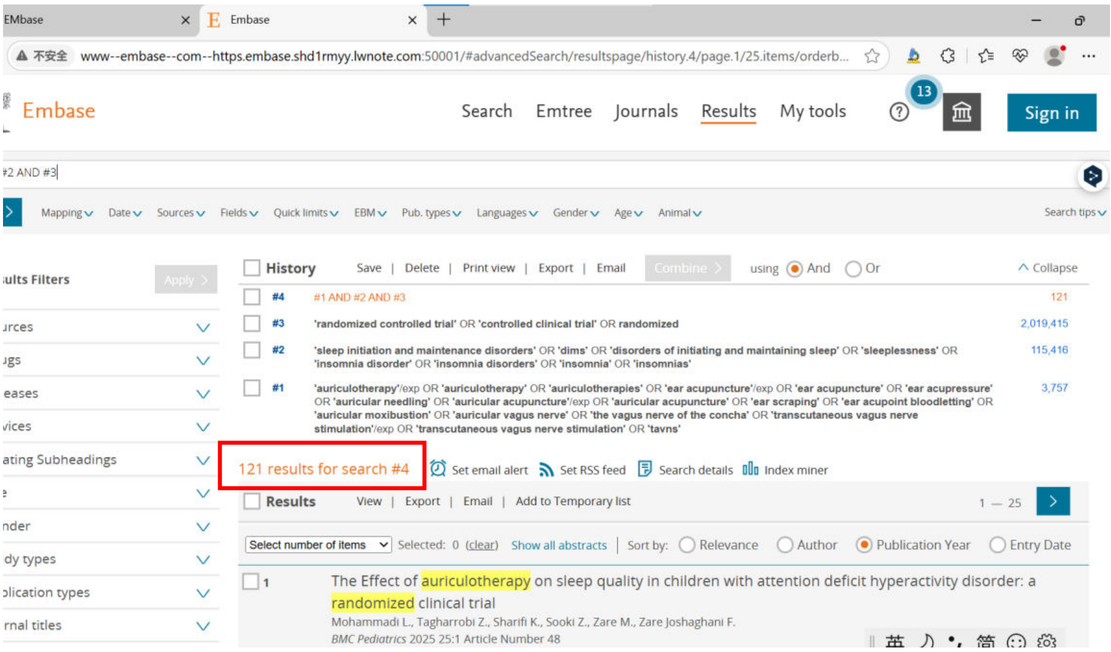

H. Cochrane Library: 115

rary × Search Manager | Cochrane Librar ×

s://www.cochranelibrary.com/advanced-search/search-manager

Search | Search manager | Medical terms (MeSH) | PICO search

Save this search | View/Share saved searches | Search help

View fewer lines | Print search history

Line	Search	Type	Results
#1	MeSH descriptor: [Auriculotherapy] explode all trees	MeSH	333
#2	(Auriculotherapies):ti,ab,kw OR (ear acupuncture):ti,ab,kw OR (ear acupressure):ti,ab,kw OR (auricular needling):ti,ab,kw OR (Auricular acupuncture):ti,ab,kw OR (Ear scraping):ti,ab,kw OR (ear acupoint bloodletting):ti,ab,kw OR (auricular moxibustion):ti,ab,kw OR (Auricular vagus nerve):ti,ab,kw OR (The vagus nerve of the concha):ti,ab,kw OR (Transcutaneous Vagus Nerve Stimulation):ti,ab,kw OR (taVNS):ti,ab,kw	Limits	2792
#3	#1 OR #2	Limits	2827
#4	MeSH descriptor: [Sleep Initiation and Maintenance Disorders] explode all trees	MeSH	4006
#5	(DIMS):ti,ab,kw OR (Disorders of Initiating and Maintaining Sleep):ti,ab,kw OR (Sleeplessness):ti,ab,kw OR (Insomnia Disorder):ti,ab,kw OR (Insomnia Disorders):ti,ab,kw OR (Insomnia):ti,ab,kw OR (Insomnias):ti,ab,kw	Limits	16323
#6	#4 OR #5	Limits	16947
#7	#3 AND #6	Limits	166
#8	((randomized controlled trial) OR (controlled clinical trial) OR (randomized)):ti,ab,kw (Word variations have been searched)	S / Limits	1475191
#9	#7 And #8	Limits	115
#10	Type a search term or use the S or MeSH buttons to compose	S / MeSH / Limits	N/A

Clear all

Highlight orphan lines

Figure S3. Risk of bias summary about each risk of bias item for each included study

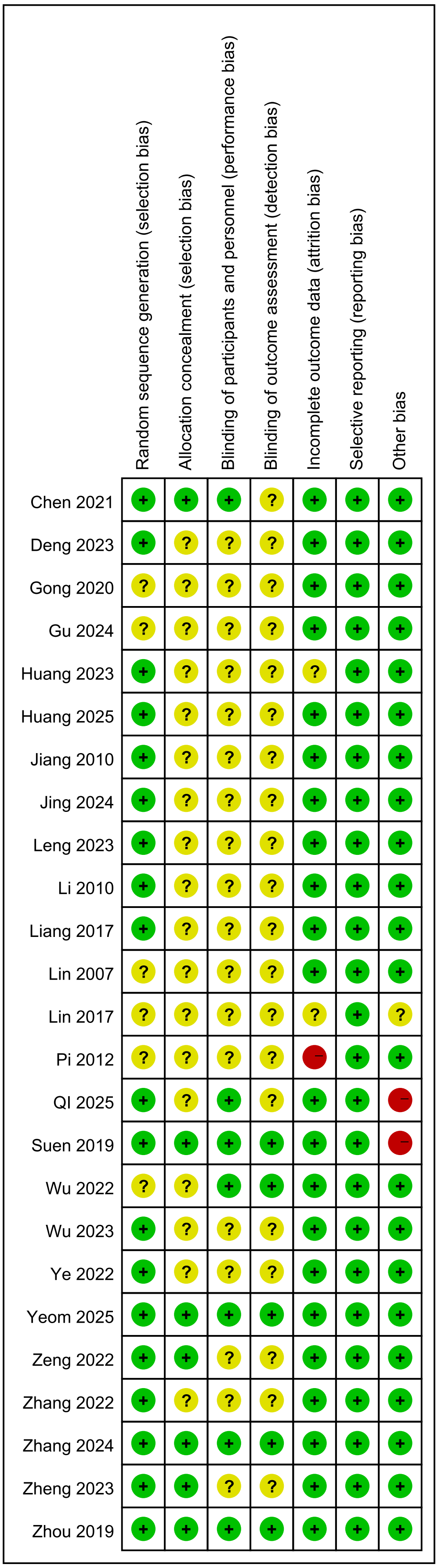

Figure S4. Inconsistency test: (A) Global inconsistency test; (B). Local inconsistency test

A. Global inconsistency test

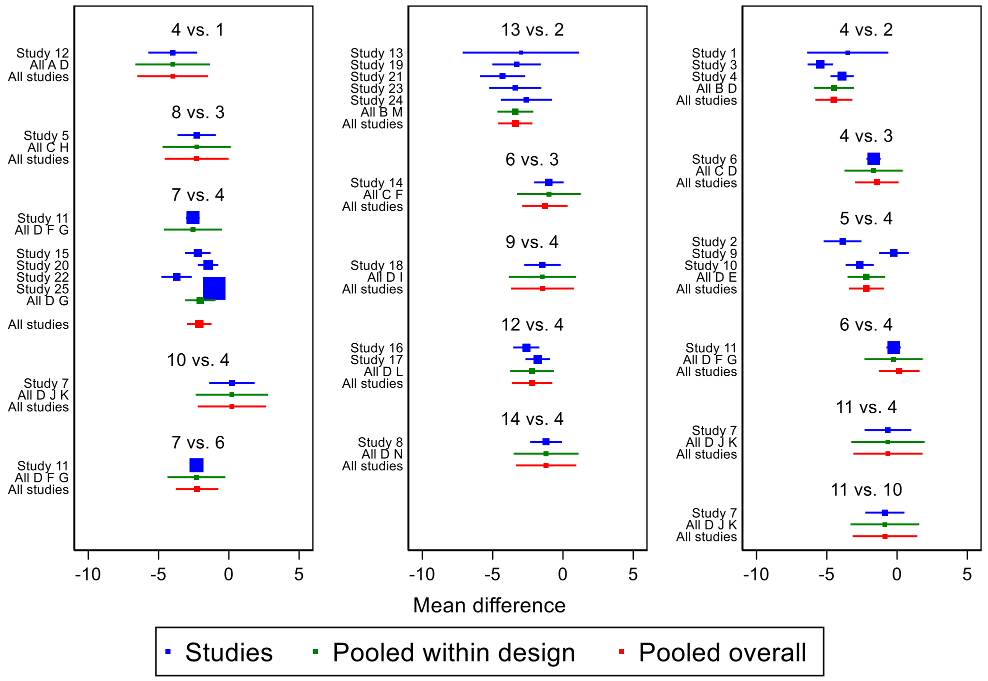

Test of consistency: chi2(2)=0.35, P=0.840

B. Local inconsistency test

Side	Direct		Indirect		Difference			tau
	Coef.	Std. Err.	Coef.	Std. Err.	Coef.	Std. Err.	P>z	
A D *	-4	1.282296	0.4652376	111.5803	-4.465238	111.5877	0.968	0.9281
B D *	-4.50056	0.6716828	-6.075329	463.1084	1.574774	463.1089	0.997	0.928096
B M *	-3.39485	0.6200645	-1.008732	999.2812	-2.386116	999.2814	0.998	0.928097
C D	-1.67	1.01024	-0.9644217	1.433784	-0.7055782	1.753944	0.687	0.976258
C F	-1	1.113062	-1.70564	1.355519	0.7056399	1.753949	0.687	0.976258
C H *	-2.29	1.160568	5.12173	1844.488	-7.41173	1844.489	0.997	0.928095
D E *	-2.18082	0.6317302	8.123516	798.4686	-10.30433	798.4688	0.99	0.928096
D F	-0.24977	0.9950635	0.7269548	1.19127	-0.9767209	1.554236	0.53	0.95975
D G *	-2.13792	0.4680672	-0.7253715	3.475252	-1.412546	3.507642	0.687	0.976251
D I *	-1.46	1.140768	7.977155	1717.699	-9.437155	1717.699	0.996	0.928095
D J *	0.22	1.24148	8.52814	2439.109	-8.30814	2439.109	0.997	0.928095
D K *	-0.65	1.257039	7.663851	2482.607	-8.31385	2482.608	0.997	0.928089
D L *	-2.19963	0.7325702	8.010901	796.7608	-10.21053	796.7612	0.99	0.928096
D N *	-1.2	1.093982	7.979815	1448.876	-9.179815	1448.876	0.995	0.928096
F G	-2.31176	1.012333	-2.179614	1.368593	-0.1321475	1.700915	0.938	0.985749
J K	.	.	.	.	.	.	.	.
