# Supplementary material for: Efficacy of auriculotherapies for primary insomnia: A systematic review and network meta-analysis
Source: Medicine (Baltimore). 2026 Apr 24;105(17):e48357. doi: 10.1097/MD.0000000000048357 (PMC13124389; doi:10.1097/MD.0000000000048357)
Supplement: Supplementary file 2 [file medi-105-e48357-s002.pdf]

Table S1. Search strategy

Database	Search strategy
PubMed	((auriculotherapy[[Mesh]) OR (auriculotherapies[Title/Abstract]) OR ("ear acupuncture"[Title/Abstract]) OR ("ear acupressure"[Title/Abstract]) OR ("auricular needling"[Title/Abstract]) OR ("auricular acupuncture"[Title/Abstract]) OR ("ear scraping"[Title/Abstract]) OR ("ear acupoint bloodletting"[Title/Abstract]) OR ("auricular moxibustion"[Title/Abstract]) OR ("auricular vagus nerve"[Title/Abstract]) OR ("the vagus nerve of the concha"[Title/Abstract]) OR ("transcutaneous vagus nerve stimulation"[Title/Abstract]) OR ("taVNS"[Title/Abstract])) AND (("sleep initiation and maintenance disorders"[Mesh]) OR ("DIMS"[Title/Abstract]) OR ("disorders of initiating and maintaining sleep"[Title/Abstract]) OR ("sleeplessness"[Title/Abstract]) OR ("insomnia disorder*"[Title/Abstract]) OR ("insomnia*"[Title/Abstract])) AND ("randomized controlled trial"[pt] OR "controlled clinical trial"[pt] OR randomized)
Web of science	TS=(auriculotherap* OR "ear acupuncture" OR "ear acupressure" OR "auricular needling" OR "auricular acupuncture" OR "ear scraping" OR "ear acupoint bloodletting" OR "auricular moxibustion" OR "auricular vagus nerve" OR "the vagus nerve of the concha" OR "transcutaneous vagus nerve stimulation" OR "taVNS") AND TS=("sleep Initiation and Maintenance Disorders" OR "DIMS" OR "disorders of initiating and maintaining sleep" OR "sleeplessness" OR "insomnia disorder*" OR "Insomnia*")
Embase	#1: 'auriculotherap*' OR 'ear acupuncture' OR 'ear acupressure' OR 'auricular needling' OR 'auricular acupuncture' OR 'ear scraping' OR 'ear acupoint bloodletting' OR 'auricular moxibustion' OR 'auricular vagus nerve' OR 'The vagus nerve of the concha' OR 'transcutaneous vagus nerve stimulation' OR 'taVNS'<br>#2: 'sleep Initiation and Maintenance Disorders' OR 'DIMS' OR 'disorders of initiating and maintaining sleep' OR 'sleeplessness' OR 'insomnia disorder*' OR 'Insomnia*'<br>#3: 'randomized controlled trial' OR 'controlled clinical trial' OR randomized<br>#4: #1 AND #2 AND #3
Cochrane library	#1: MeSH descriptor: [Auriculotherapy] explode all trees<br>#2: (auriculotherapies):ti,ab,kw OR (ear acupuncture):ti,ab,kw OR (ear acupressure):ti,ab,kw OR (auricular needling):ti,ab,kw OR (auricular acupuncture):ti,ab,kw OR (ear scraping):ti,ab,kw OR (ear acupoint bloodletting):ti,ab,kw OR (auricular moxibustion):ti,ab,kw OR (auricular vagus nerve):ti,ab,kw OR (the vagus nerve of the concha):ti,ab,kw OR (transcutaneous vagus nerve stimulation):ti,ab,kw OR (taVNS):ti,ab,kw<br>#3: #1 OR #2<br>#4: MeSH descriptor:[sleep initiation and maintenance disorders] explode all trees<br>#5: (DIMS):ti,ab,kw OR (disorders of initiating and maintaining sleep):ti,ab,kw OR (Sleeplessness):ti,ab,kw OR (insomnia disorder*):ti,ab,kw OR (insomnia*):ti,ab,kw<br>#6: #4 OR #5<br>#7: (randomized):ab<br>#8: #3 AND #6 AND #7
CNKI	主题=（耳穴 + 耳针 + 耳部刮痧 + 耳尖放血 + 耳部放血 + 耳灸 + 耳揿针 + 耳迷走神经 + 耳部迷走神经 + 耳穴迷走神经 + 耳甲迷走神经）AND 主题=（失眠 + 睡眠障碍 + 不寐）AND 篇关摘=（随机）

Wanfang	主题=（耳穴 OR 耳针 OR 耳迷走神经 OR 耳部刮痧 OR 耳部放血 OR 耳尖放血 OR 耳灸 OR 耳揿针 OR 耳部迷走神经 OR 耳穴迷走神经 OR 耳甲迷走神经）AND 主题=（失眠 OR 睡眠障碍 OR 不寐）AND 摘要=（随机）
VIP	题名或关键词=（耳穴 OR 耳针 OR 耳迷走神经 OR 耳部刮痧 OR 耳部放血 OR 耳尖放血 OR 耳灸 OR 耳揿针 OR 耳部迷走神经 OR 耳穴迷走神经 OR 耳甲迷走神经）AND 题名或关键词=（失眠 OR 睡眠障碍 OR 不寐）AND 摘要=（随机）
CBM	常用字段=（耳穴 OR 耳针 OR 耳迷走神经 OR 耳部刮痧 OR 耳部放血 OR 耳尖放血 OR 耳灸 OR 耳揿针 OR 耳部迷走神经 OR 耳穴迷走神经 OR 耳甲迷走神经）AND 常用字段=（失眠 OR 睡眠障碍 OR 不寐）AND 摘要=（随机）
